# Uptake of Goal-Directed Therapies in a Multidisciplinary and Interdisciplinary Cardiology-Renal-Endocrine Clinic: A Research Letter

**DOI:** 10.1177/20543581251380509

**Published:** 2025-10-05

**Authors:** Jean-Philippe Ouimet, Mai Mohsen, Huajing Ni, Alanna Weisman, Jacob A. Udell, David Z.I. Cherney

**Affiliations:** 1 Division of Nephrology, University Health Network, Toronto, ON, Canada; 2 Temerty Faculty of Medicine, University of Toronto, ON, Canada; 3 Division of Endocrinology & Metabolism, Department of Medicine, University of Toronto, ON, Canada; 4 Cardiovascular Division, Peter Munk Cardiac Centre, Toronto General Hospital and Women’s College Hospital, University of Toronto, ON, Canada; 5 Institute for Clinical Evaluative Sciences, Toronto, ON, Canada

**Keywords:** diabetes mellitus, chronic kidney disease, cardiovascular disease, interdisciplinary clinic, cardiovascular-kidney-metabolic syndrome

## Abstract

The Cardiac and Renal Endocrine (C.a.R.E) clinic is an interdisciplinary clinic offering integrated care to complex patients with cardiovascular-kidney-metabolic syndrome. Previous data from the clinic demonstrated improvements in clinical parameters but provided limited information on the indicated usage of evidence-based therapies. We aimed to update baseline characteristics and clinical data of the C.a.R.E clinic cohort, with added information on the uptake of therapies and reasons for therapy non-use. We performed a retrospective chart review of patients seen in the C.a.R.E clinic between July 2014 and July 2024 with at least two documented clinic visits. Data from the first and last visits were compared to evaluate treatment uptake and changes in clinical parameters. A total of 125 patients met our inclusion criteria. There were significant improvements in low-density lipoprotein levels (1.61 mmol/L for last visit vs 1.82 mmol/L for first visit), blood pressure (BP) measurements, (median systolic BP of 126 mm Hg vs 130 mm Hg and median diastolic BP of 72 mm Hg vs 76 mm Hg), and proportion of patients achieving BP targets (60.0% vs 44.8%). Uptake of therapies significantly increased, including sodium-glucose cotransporter-2 inhibitors (59.2% vs 24.0%) and glucagon-like peptide-1 receptor agonists (30.4% vs 9.6%). The use of finerenone also increased (22.7% vs 3.0%) among the 66 patients whose last visit occurred after Health Canada approved finerenone. The C.a.R.E clinic demonstrates potential to improve therapy uptake and clinical outcomes in patients with cardiovascular-kidney-metabolic syndrome. However, consistent clinical documentation and innovative strategies are needed to further enhance the adoption of evidence-based therapies.

## Abrégé

La clinique C.a.R.E (Cardiac and Renal Endocrine) est une clinique interdisciplinaire proposant une prise en charge intégrée des patients complexes présentant un syndrome cardio-rénal métabolique. Les données antérieures issues de la clinique ont montré des améliorations des paramètres cliniques, mais ont fourni peu d’informations sur l’utilisation indiquée de thérapies fondées sur des données probantes. Notre objectif était de mettre à jour les caractéristiques initiales et les données cliniques de la cohorte de la clinique C.a.R.E, en y ajoutant des informations sur l’adoption des thérapies, ainsi que sur les raisons de leur non-utilisation. Nous avons procédé à une analyse rétrospective des dossiers médicaux des patients pris en charge à la clinique C.a.R.E. et retenu les patients avec au moins deux visites documentées entre juillet 2014 et juillet 2024. Les données de la première et de la dernière visite ont été comparées pour évaluer la prise en charge du traitement et les modifications des paramètres cliniques. En tout, 125 patients répondaient aux critères d’inclusion. Des améliorations significatives ont été observées dans les taux de lipoprotéines de basse densité (1,61 mmol/l à la dernière visite c. 1,82 mmol/l à la première visite), les mesures de la pression artérielle (PA), (PA systolique médiane : 126 mm Hg c. 130 mm Hg; PA diastolique médiane : 72 mm Hg c. 76 mm Hg) et la proportion de patients ayant atteint les cibles de PA (60 % c. 44 %). On a noté une augmentation significative de la prise des thérapies, en particulier pour les inhibiteurs du cotransporteur sodium-glucose de type 2 (59 % c. 24,0 %) et les agonistes des récepteurs du peptide-1 de type glucagon (30 % c. 9,6 %). On a également observé une augmentation de la prise de finérénone (22,7 % c. 3,0 %) chez les 66 patients dont la dernière visite avait eu lieu après l’approbation du médicament par Santé Canada. La clinique C.a.R.E. démontre un potentiel d’accroissement de l’utilisation des thérapies et d’amélioration des issues cliniques chez les patients présentant un syndrome cardio-rénal métabolique. Une documentation clinique cohérente et des stratégies novatrices sont toutefois nécessaires pour accroître davantage l’utilisation des thérapies validées par les données probantes.

## Introduction

In 2022, Dubrofsky et al^1^ published the first data on the Cardiac and Renal Endocrine (C.a.R.E.) clinic, a multidisciplinary and interdisciplinary care model based at the Toronto General Hospital. The clinic comprises a team of specialists and allied health professionals, focused on improving cardiovascular-kidney-metabolic (CKM) health by implementing evidence-based care.

The initial analysis demonstrated improvements in clinical parameters including low-density lipoprotein (LDL) cholesterol, hemoglobin A1c (HbA1c), and blood pressure (BP), along with increased uptake of evidence-based therapies.^1^ However, the uptake of sodium-glucose cotransporter-2 inhibitors (SGLT2i) and glucagon-like peptide-1 receptor agonists (GLP-1RA) remained low at 35.1% and 13.5%, respectively, while the non-steroidal mineralocorticoid receptor antagonist (nsMRA), finerenone, had not yet received Health Canada approval.

Since the original publication, new evidence has emerged leading to expanded indications for cardiorenal therapies including the use of semaglutide for nephroprotection.^2^ Given the evolving treatment landscape across the spectrum of CKM disease, we aimed to evaluate the uptake of evidence-based therapies within the C.a.R.E clinic and assess their impact on clinical parameters.

## Methods

We conducted a retrospective chart review of patients treated in the C.a.R.E clinic. The inclusion criteria have been previously described.^1^ Briefly, patients with diabetes and coexisting chronic kidney disease (CKD G1-G4) and/or cardiovascular disease (CVD) are referred to the clinic by their primary care or specialist physician for integrated management, while continuing to follow-up with their primary physician. We included all patients with at least two clinic visits to establish a before and after timeframe. For each visit, we collected demographic, clinical and medication data using paper charts and electronic health records (EHR). Clinical data included laboratory results, body mass index (BMI), BP, and vascular complications of diabetes.

We recorded medication use at each visit (including renin-angiotensin-aldosterone system inhibitors [RAASi], statins, SGLT2i, GLP-1RA, and nsMRA), and estimated each patient’s eligibility for treatment at their last clinic visit. We assessed eligibility for statins and RAASi using clinical practice guidelines from Diabetes Canada.^3,4^ As indications for SGLT2i, GLP-1RA and nsMRA evolved over the study period, we assessed eligibility for these therapies using eligibility criteria from clinical trials that were published at the time each patient was evaluated (Supplementary Table 1). Since finerenone was approved by Health Canada on October 14, 2022, we created a sub-group of patients whose last clinic visit occurred after this date to more accurately reflect nsMRA uptake.

To assess care performance within the clinic, we investigated reasons for non-use of therapies in patients with clear indications and no contraindications. We identified six main categories for medication non-use including (1) treatment-emergent adverse events, (2) coverage limitations, (3) patient refusal to initiate therapy, (4) patient enrollment in a clinical trial where they may be receiving the same class of medication, (5) provider’s future plan to initiate therapy, and (6) unclear. Medication non-use was classified as “unclear” when no other documented explanation was available, and the rationale for decision making could not be inferred from the patient’s clinical history.

Data was cleaned manually with cross-validation by two reviewers (J-PO & MM). No duplicates or outliers were identified. Complete case analysis was applied to handle the missing data. To calculate statistical significance between the paired data from the first and last visits, we used the Wilcoxon signed rank test and McNemar test for continuous and categorical variables, respectively. All statistical analyses evaluating treatment effects used a 5% significance level and were two-sided. All analyses were performed using SPSS v29 for Mac (IBM Corp., Armonk, NY, USA).

## Results

Between July 2014 and July 2024, 125 patients met our inclusion criteria. At baseline (first clinic visits), the median age was 67 years, 65.6% were male, and the mean follow-up duration was 41.1 months (standard deviation of 36.0 months). Baseline characteristics of the study cohort are shown in Supplementary Table 2.

BP and LDL levels were significantly reduced in the last clinic visits compared to baseline, although changes in BMI, HbA1c, and albuminuria levels (measured as urine albumin-creatinine ratio) were not significant (Supplementary Table 3). The uptake of SGLT2i, GLP-1RA, and finerenone significantly increased from baseline, whereas the use of RAASi and statins did not change significantly (Supplementary Table 3 and Supplementary Figure 1).

**Figure 1.**
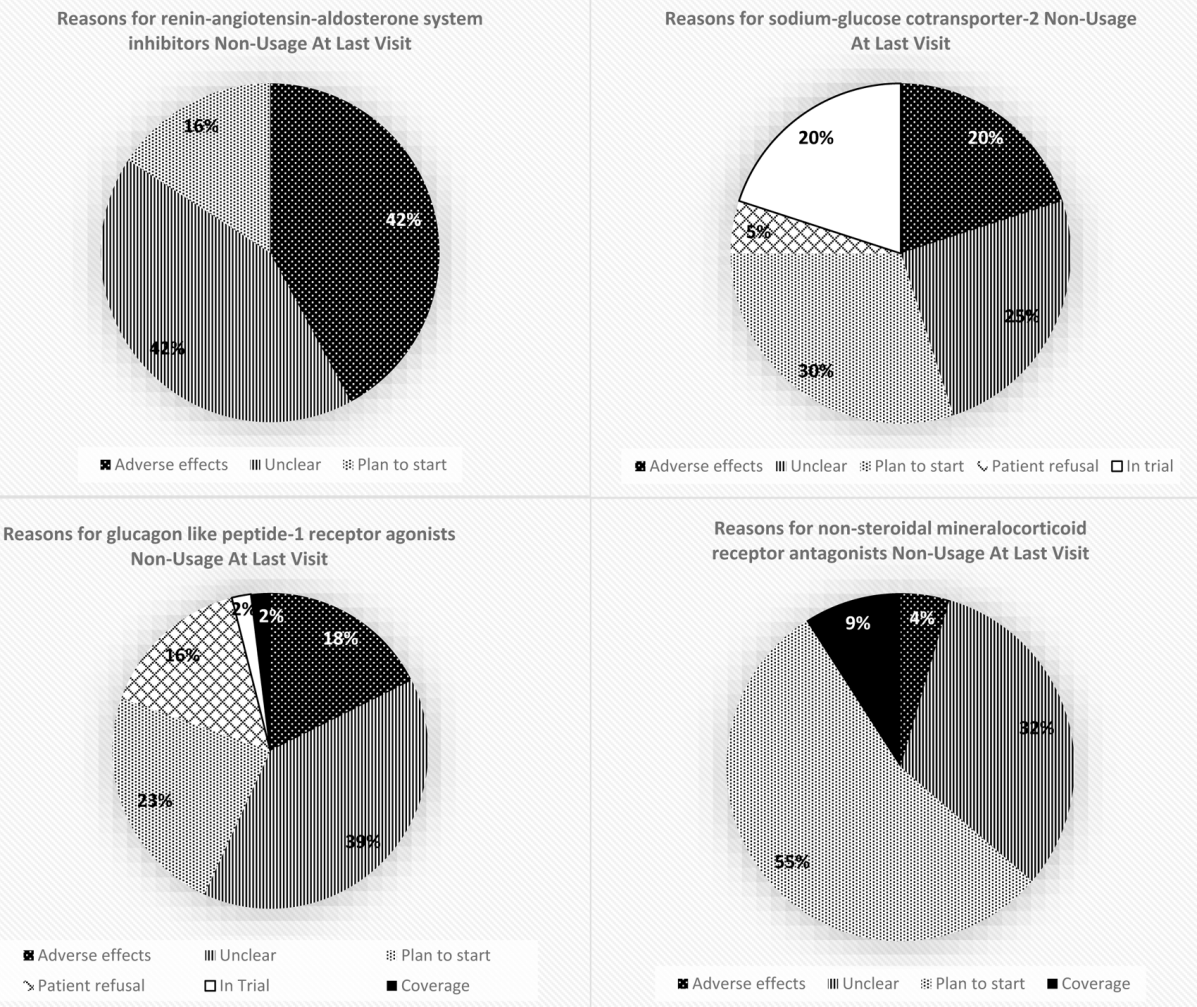
Reasons for non-usage of evidence-based therapies in the C.a.R.E clinic. *Note*. Percentage uptake pertains to the groups who have indications for evidence- based medical therapies. Percentage uptake for finerenone refers to usage in a pre-defined sub-group of 66 patients.

In the overall cohort, 92.8% had an indication for RAASi, 76.0% for SGLT2i, and 72.0% for GLP-1RA. Among the finerenone sub-group (n = 66), 56.1% had an indication for finerenone. Primary reasons for medication non-use among indicated patients are shown in Figure 1. Those included unclear (RAASi: 42%, GLP-1RA: 39%, finerenone: 32%, SGLT2i: 25%), planned treatment initiation (finerenone: 55%, SGLT2i: 30%, GLP-1RA: 23%, RAASi: 16%), and treatment-emergent adverse effects (RAASi: 42%, SGLT2i: 20%, GLP-1RA: 18%, finerenone: 4%). Adverse effects included hypotension, hyperkalemia, and acute kidney injury with RAASi; urinary symptoms, acute kidney injury, and euglycemic diabetic ketoacidosis with SGLT2i; gastrointestinal effects and weight loss with GLP-1RA; and hyperkalemia with finerenone.

## Discussion

We assessed the uptake of evidence-based therapies and their impact on clinical parameters among patients with CKM syndrome treated at a multidisciplinary clinic. Consistent with previous findings,^1^ our analysis showed improvement in clinic BP readings between the first and last clinic visits, corresponding to an increase in the proportion of patients meeting the recommended BP target. However, BMI did not change significantly from baseline, highlighting weight management as an opportunity for ongoing quality improvement in the clinic. Similarly, HbA1c levels were not significantly reduced, likely because baseline glycemic control was close to target or that older/frail patients had less stringent targets than the general population.

Statin use was already high at baseline although we found a significant reduction in LDL level, likely due to optimization of the statin dose according to tolerance and the use of additional therapy (ezetimibe or PCSK9 inhibitors) during the follow-up period. RAASi use did not change throughout the study period, also likely because of high RAASi use at baseline and optimization of BP through other medication classes.

Uptake of SGLT2i was higher in our study, with 59.2% of patients on SGLT2i at the last visit versus 35.1% in the initial analysis. This increase may be explained by the expanded indication for SGLT2i to include an estimated glomerular filtration rate (eGFR) down to 20 ml/min/1.73m^2^.^5^ Other explanations for this increase include expanding coverage and greater familiarity with prescription over time.

GLP-1RA use increased to 30.4% at the last visit compared to 13.5% in the initial analysis. Many patients referred to the C.a.R.E clinic are frail with multiple comorbidities, making weight loss a less desired effect of GLP-1RA. Other agents which are weight neutral may be preferred and prioritized. However, 39% of indicated patients were not using GLP-1RA without clear justification. This finding reflects a need for consistent documentation for non-initiation of treatment and may also reflect provider uncertainty with GLP-1RA use, particularly for nephroprotection. Results of the FLOW^2^ trial and findings from recent meta-analyses^6^ are expected to promote the uptake of GLP-1RA over time.

The use of finerenone increased from 3.0% to 22.7% among the 66 patients whose last clinic visit occurred after Health Canada approval. Slower uptake of finerenone is unsurprising given its initial coverage limitations (listed in the Ontario Drug Benefit formulary in March 2024). Since the primary reason for finerenone non-use was a plan to initiate therapy (55%), it is likely that longer follow-up periods will show increased uptake.

Although the combined use of RAASi, SGLT2i, GLP-1RA, and nsMRA is yet to be prospectively studied in people with CKD, recent analyses suggest additive benefits on cardio-renal-metabolic outcomes with combination therapy.^7-9^ In our finerenone sub-group, 7 out of 66 patients were receiving all four medication classes, a number that may increase with the evolving evidence.^10^

A key strength of this study is its longitudinal design, which tracks a cohort of complex patients over time, demonstrating real-world trends in medication use and reasons for non-use. However, our study is limited by its retrospective design, and the lack of a comparator cohort not treated in this clinic. Additionally, there was considerable heterogeneity in the time interval between clinic visits ranging from months to years, however this variable was not collected. Furthermore, publication of new evidence and commercialization of new therapies contributed to significant practice changes during this period. These factors prevent us from drawing causal inferences regarding the observed clinical improvements.

## Conclusions

We have updated previously published data on medication use and clinical outcomes in patients with CKM syndrome. Our findings suggest that the C.a.R.E clinic improves uptake of evidence-based therapies and helps patients reach clinical targets. However, consistent documentation in EHR regarding non-initiation of therapies, as well as innovative strategies are needed to promote future consideration and uptake.

Future studies investigating the impact of multidisciplinary clinics, across diverse geographic settings with varying availability of subspecialties, are needed to test the generalizability of this care model. For example, Scarborough Health Network recently launched a multidisciplinary clinic based on a similar model, where a nephrologist and endocrinologist jointly assess patients at each visit. Prospective evaluations of such clinics may have important implications for care.

## Supplemental Material

sj-docx-1-cjk-10.1177_20543581251380509 – Supplemental material for Uptake of Goal-Directed Therapies in a Multidisciplinary and Interdisciplinary Cardiology-Renal-Endocrine Clinic: A Research Letter Supplemental material, sj-docx-1-cjk-10.1177_20543581251380509 for Uptake of Goal-Directed Therapies in a Multidisciplinary and Interdisciplinary Cardiology-Renal-Endocrine Clinic: A Research Letter by Jean-Philippe Ouimet, Mai Mohsen, Huajing Ni, Alanna Weisman, Jacob A. Udell and David Z.I. Cherney in Canadian Journal of Kidney Health and Disease
