# Supplementary material for: Uptake of Goal-Directed Therapies in a Multidisciplinary and Interdisciplinary Cardiology-Renal-Endocrine Clinic: A Research Letter
Source: Can J Kidney Health Dis. 2025 Oct 5;12:20543581251380509. doi: 10.1177/20543581251380509 (PMC12497978; doi:10.1177/20543581251380509)
Supplement: sj-docx-1-cjk-10.1177_20543581251380509 – Supplemental material for Uptake of Goal-Directed Therapies in a Multidisciplinary and Interdisciplinary Cardiology-Renal-Endocrine Clinic: A Research Letter [file sj-docx-1-cjk-10.1177_20543581251380509.docx]

# **Supplementary Tables**

**Supplementary Table 1: Clinical trial publication timeline and Health Canada approval dates used to assess treatment eligibility in the C.a.R.E clinic cohort**

| **Clinical trial publication date** | **Clinical trial** | **Eligibility criteria applied in our study** |
| --- | --- | --- |
| August 18, 2015 | SCALE-Diabetes (NCT01272232) | GLP-1RA in adults with type 2 diabetes and BMI ≥27.0 |
| November 26, 2015 | EMPA-REG OUTCOME  (NCT01131676) | SGLT2i in adults with type 2 diabetes and ASCVD or HF and eGFR≥30 ml/min/1.73m² |
| July 28,  2016 | LEADER  (NCT01179048) | GLP-1RA in adults with type 2 diabetes and ASCVD |
| November 10, 2016 | SUSTAIN-6  (NCT01720446) | GLP-1RA in adults with type 2 diabetes and ASCVD |
| April 14,  2019 | CREDENCE  (NCT02065791) | SGLT2i in adults with type 2 diabetes, CKD with albuminuria, and eGFR ≥30 ml/min/1.73m² |
| September 24, 2020 | DAPA-CKD  (NCT03036150) | SGLT2i in adults with or without type 2 diabetes, CKD with albuminuria, and eGFR ≥ 25 ml/min/1.73m² |
| October 23, 2020 | FIDELIO-DKD  (NCT02540993) | Finerenone in adults with type 2 diabetes, CKD with albuminuria, serum potassium of ≤ 4.8 mmol/L, eGFR ≥ 25 ml/min/1.73m², and treated with an ACE inhibitor or an ARB |
| August 28, 2021 | FIGARO-DKD  (NCT02545049) |  |
| November 4, 2022 | EMPA-KIDNEY  (NCT03594110) | SGLT2i in adults with or without diabetes, CKD and eGFR ≥ 20 ml/min/1.73m² |
| May 24,  2024 | FLOW  (NCT03819153) | GLP-1RA in adults with type 2 diabetes, CKD with albuminuria and eGFR ≥ 25 ml/min/1.73m² |

BMI: Body mass index; CKD: Chronic kidney disease; HF: Heart failure; ASCVD: Atherosclerotic cardiovascular disease; GLP-1RA: Glucagon-like peptide-1 receptor agonists; SGLT2i: Sodium-glucose cotransporter-2 inhibitors; eGFR: estimated glomerular filtration rate; ACE: Angiotensin-converting enzyme; ARB: Angiotensin receptor blocker.

Treatment eligibility assessment for GLP-1RA in 2014 was determined using the Canadian Diabetes Association Clinical Practice Guidelines (2013); Health Canada approval date for finerenone: October 14, 2022.

**Supplementary Table 2: C.a.R.E clinic cohort baseline characteristics**

| **Baseline characteristics (n=125)** | **First visit data** |
| --- | --- |
| Age, year (IQR) | 67 (54, 75) |
| No. (%) male | 82 (65.6) |
| No. of clinic visits (IQR) | *Not in dataset* |
| Time between first and last visit, days (IQR) | 847 (346, 2075) |
| No. (%) with chronic kidney disease | 121 (96.8) |
| G1 (eGFR ≥ 90) | 14 (11.2) |
| G2 (eGFR 60-89) | 20 (16.0) |
| G3 (eGFR 30-59) | 71 (56.8) |
| G4 (eGFR 15-29) | 18 (14.4) |
| G5 (eGFR < 15) | 0 |
| Normal to mildly increased albuminuria (A1) - No. (%) | 18 (23.7) |
| Moderately increased albuminuria (A2) - No. (%) | 31 (40.8) |
| Severely increased albuminuria (A3) - No. (%) | 27 (35.5) |
| No. (%) with retinopathy | 39 (31.2) |
| No. (%) with neuropathy | 35 (28) |
| No. (%) with coronary artery disease | 39 (31.2) |
| No. (%) with peripheral vascular disease | 17 (13.6) |
| No. (%) with stroke/transient ischemic attack | 15 (12) |

Continuous data are presented as median (IQR) or mean (SD) depending on the distribution; categorical data are presented as No. (%)

**Supplementary Table 3: Differences in clinical parameters observed between the first and last clinic visits**

| **Variables** | **First visit data** | **Last visit data** | **P-value** | **Missing data (FV; LV)** |
| --- | --- | --- | --- | --- |
| Body mass index, kg/m^2^ | 28.8 (24.9, 33.3) | 27.9 (24.3, 32.3) | 0.068 | 18 (14.4%); 27 (21.6%) |
| Systolic blood pressure, mm Hg | 129.5 (120, 148.5) | 126 (115, 138) | 0.033 | 15 (12.0%); 18 (14.4%) |
| Diastolic blood pressure, mm Hg | 76 (70, 83) | 72 (66, 79) | 0.006 | 14 (11.2%); 19 (15.2%) |
| Blood pressure ≤ 130/80 No. (%) | 56 (44.8) | 75 (60.0) | 0.019 |  |
| Hemoglobin A1C, % | 7.3 (6.57, 8.33) | 7.1 (6.4, 8.2) | 0.156 | 3 (2.4%); 2 (1.6%) |
| Estimated glomerular filtration rate, mL/min/1.73 m^2^ | 45 (34, 61) | 40 (29, 57) | < 0.001 | 2 (1.6%); 2 (1.6%) |
| Albuminuria, mg/mmol | 17.7 (3.1, 51.0) | 15.6 (3.5, 66.8) | 0.369 | 49 (39.2%); 23 (18.4%) |
| Low-density lipoprotein, mmol/L | 1.82 (1.40, 2.41) | 1.61 (1.25, 2.07) | 0.005 | 14 (11.2%); 13 (10.4%) |
| Aspirin and/or clopidogrel use—No. (%) | 59 (47.2) | 55 (44.0) | 0.07 |  |
| Renin-angiotensin-aldosterone system inhibitor use—No. (%) | 98 (78.4) | 96 (76.8) | 0.876 |  |
| Statin use—No. (%) | 101 (80.8) | 111 (88.8) | 0.067 |  |
| Sodium-glucose cotransporter-2 inhibitor use—No. (%) | 30 (24.0) | 74 (59.2) | < 0.001 |  |
| Glucagon-like peptide-1 receptor agonist use—No. (%) | 12 (9.6) | 38 (30.4) | < 0.001 |  |
| Non-steroidal mineralocorticoid receptor antagonist use—No. (%) | 2 (3.0) | 15 (22.7) | 0.002 |  |

Continuous data are presented as median (IQR) or mean (SD) depending on the distribution; categorical data are presented as No. (%)

# **Supplementary Figure**

**Supplementary Figure 1: Percentage uptake of evidence-based therapies in the C.a.R.E Clinic**


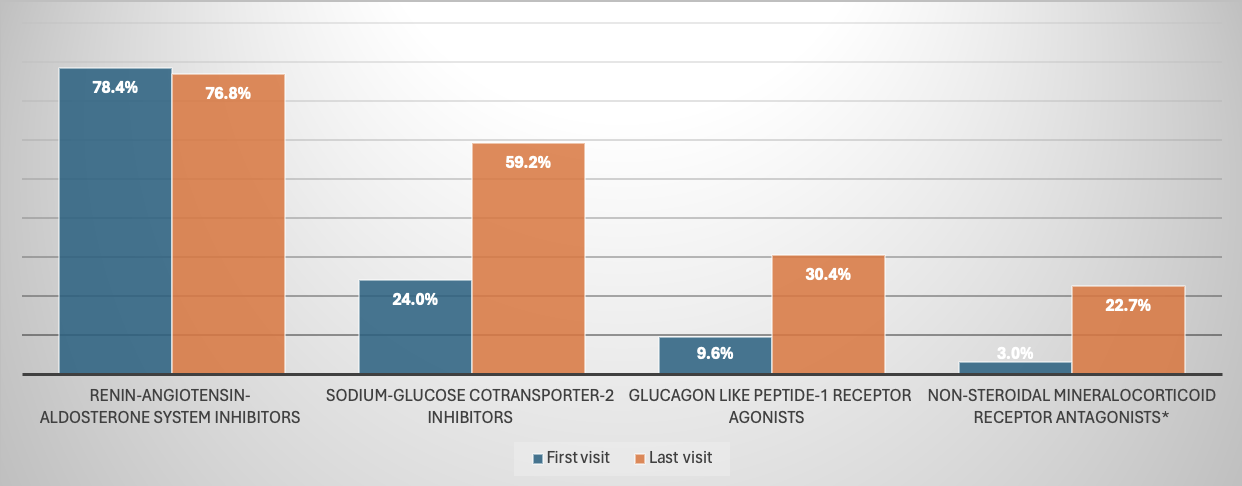


Percentage uptake pertains to all patients

Percentage uptake for finerenone refers to usage in pre-defined subgroup of 66 patients
